# Collagen Type V alpha 1 chain and alpha‐actinin‐3 variants predict knee ligament injury risk in professional football players

**DOI:** 10.1002/jeo2.70724

**Published:** 2026-04-20

**Authors:** Yoshitomo Saita, Nanako Yamamoto, Eri Miyamoto‐Mikami, Takaya Ohtaki, Hidenori Izawa, Yoshifumi Fukushima, Muneaki Ishijima, Noriyuki Fuku

**Affiliations:** ^1^ Department of Orthopaedics, Faculty of Medicine Juntendo University Tokyo Japan; ^2^ Department of Medical IWAKI Sports Club Fukushima Japan; ^3^ Graduate School of Health and Sports Science Juntendo University Chiba Japan; ^4^ Department of Cardiovascular Biology and Medicine Juntendo University Tokyo Japan

**Keywords:** anterior cruciate ligament, genetics, injury prevention, medial collateral ligament

## Abstract

**Purpose:**

To investigate whether polymorphisms in collagen Type V alpha 1 chain (COL5A1), actinin alpha 3 (ACTN3) and angiotensin‐converting enzyme (ACE) are associated with susceptibility to anterior cruciate ligament (ACL) and medial collateral ligament (MCL) injuries in professional football players.

**Methods:**

Between 2017 and 2025, 122 male professional football players were enroled. Genotyping was performed for COL5A1 rs12722, COL5A1 rs10628678 (formerly rs71746744), ACTN3 rs1815739 and ACE rs4341. Players were classified based on the history of ACL/MCL injuries and prospectively monitored injuries. Logistic regression was used to calculate odds ratios and 95% confidence intervals.

**Results:**

Forty‐three players sustained ACL or MCL injuries (15 before and 28 after team enrolment), including 17 ACL injuries. The COL5A1 rs10628678 dominant model (–/–+AGGG/– vs. AGGG/AGGG) was associated with an increased risk of overall knee ligament injury. The ACTN3 rs1815739 recessive model (XX vs. RR + RX) was associated with overall ligament and ACL injuries. Combined genotype analysis revealed that players with COL5A1 rs10628678 –/– and ACTN3 rs1815739 XX had the highest risk. When a simplified combined risk variable was defined (COL5A1 rs10628678 AGGG/– or –/– plus ACTN3 rs1815739 XX), this group accounted for 18.9% of the cohort and showed a significantly higher prevalence of ligament (70.7% vs. 29.3%) and ACL (30.4% vs. 10.1%) injuries. Logistic regression confirmed an independent threefold increase in risk.

**Conclusion:**

COL5A1 and ACTN3 variants may be associated with susceptibility to knee ligament injuries. Notably, this study suggests that both ligament‐related (COL5A1) and muscle function‐related genetic characteristics (ACTN3) may jointly influence injury risk. A simplified combined risk model identified nearly one‐fifth of players as genetically high‐risk, with an approximately threefold higher likelihood of ligament injury. These findings suggest that a single genetic test may help identify athletes at elevated risk who could potentially benefit from targeted preventive neuromuscular training strategies.

**Level of Evidence:**

Level IV.

## Graphical abstract

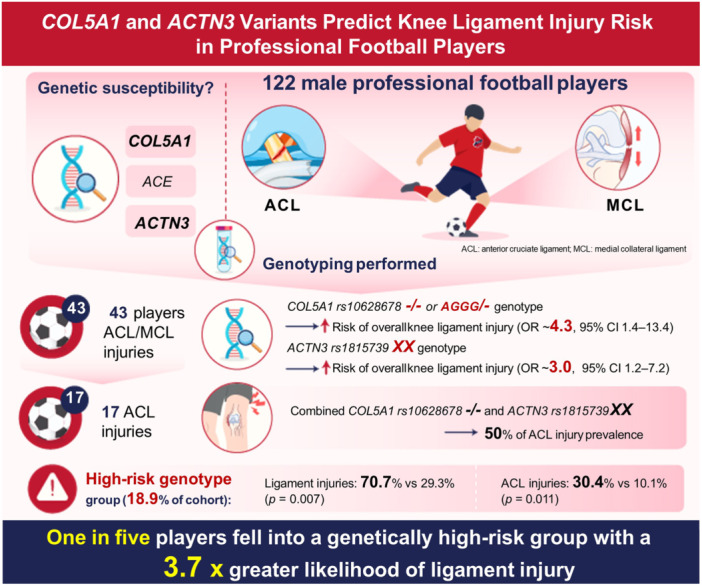

**Abbreviations**
ACE: angiotensin‐converting enzyme gene
ACL: anterior cruciate ligament
ACTN3: alpha‐actinin‐3 gene
CI: confidence interval
COL5A1: collagen Type V alpha 1 chain gene
DNA: deoxyribonucleic acid
FDR: false discovery rate
MCL: medial collateral ligament
OR: odds ratio
PCR: polymerase chain reaction
SD: standard deviation
SNP: single‐nucleotide polymorphism

## INTRODUCTION

Knee ligament injuries are common in professional football and frequently affect the medial collateral ligament (MCL) and anterior cruciate ligament (ACL) [10]. Although MCL injuries occur more frequently, ACL injuries are particularly devastating because of their severity, prolonged rehabilitation, and high risk of long‐term sequelae [4]. Therefore, identifying risk factors for these injuries is crucial for developing effective prevention strategies in elite athletes [12].

Genetic predisposition has recently emerged as a potential contributor to ligament and tendon injuries [15]. Collagen Type V alpha 1 chain gene (COL5A1), which encodes Type V collagen, has been extensively investigated and is associated with musculoskeletal soft tissue injuries, including ligament ruptures [3]. However, evidence regarding its relationship with joint flexibility is conflicting. For instance, Miyamoto‐Mikami et al. reported that COL5A1 polymorphisms were not significantly associated with flexibility in Japanese athletes [11].

The alpha‐actinin‐3 (ACTN3) gene encodes α‐actinin‐3, a protein expressed in fast‐twitch muscle fibres. The R577X polymorphism results in a null genotype (XX), which is associated with altered muscle function [17]. Recent evidence shows that the XX genotype may increase the risk of non‐contact sports injuries, potentially because of reduced muscle power and impaired dynamic joint stabilization [23]. Epidemiological studies have further revealed that greater muscle strength and lean mass are associated with a reduced risk of ligament injuries [9], likely because enhanced neuromuscular capacity improves dynamic joint stabilization and reduces mechanical loading during athletic activities. Given that ACTN3 is closely associated with muscle power and mass, analysis of its variants is biologically justified when investigating genetic susceptibility to ligament injuries.

Similarly, the angiotensin‐converting enzyme (ACE) gene insertion/deletion (I/D) polymorphisms have been widely studied in athletic performance and injury [14]. Although primarily associated with endurance and power phenotypes, ACE may also influence susceptibility to musculoskeletal injury through vascular and tissue remodelling pathways [21]. Consequently, although ACTN3 and ACE have not been directly linked to ligament rupture in previous studies, both genes are strongly associated with neuromuscular function and tissue adaptation, suggesting a plausible indirect contribution to ligament injury susceptibility.

To date, most genetic association studies have examined single candidate genes in isolation [18], and few have explored the interactions among multiple polymorphisms in the context of knee ligament injuries [19]. Such interactions may provide additional insights into individual susceptibility, especially in high‐risk populations, such as professional football players [20]. Therefore, this study aimed to investigate whether polymorphisms in COL5A1, ACTN3 and ACE are associated with susceptibility to ACL and MCL injuries in professional football players, with particular focus on combined genotype effects.

Specifically, we focused on polymorphisms in three genes: COL5A1, a key collagen gene directly implicated in connective tissue integrity; and ACTN3 and ACE, genes that influence muscle power, vascular regulation and tissue recovery. This combined approach was designed to assess both the structural and functional aspects of knee ligament vulnerability.

We hypothesized that COL5A1, ACTN3 and ACE polymorphisms, individually or in combination, are associated with the risk of knee ligament injury in professional football players. To test this hypothesis, we conducted a genetic association study in a homogeneous cohort of Division 2 professional football players, focusing on ACL and MCL injuries as clinically important outcomes.

## MATERIALS AND METHODS

### Study design and participants

This retrospective cohort study included professional male football players from a single Division 2 team in Japan between 2017 and 2025. A total of 122 players (mean age 22.1 ± 2.2 years), including eight goalkeepers, were enroled. Genetic testing was performed upon team enrolment. Data on the history of ACL or MCL injury of the players before enrolment, as well as subsequent injuries during their professional career, were obtained from club medical records.

Anthropometric and physical performance data, including height, body weight, body fat percentage, sit‐and‐reach test results, sit‐and‐reach/height ratio and vertical jump, were obtained during routine medical check‐ups (Table 1).

**Table 1:** Characteristics of the participants (*n* = 122, all male professional football players).

Variable	Mean ± SD	Range
Age (years)	22.1 ± 2.2	18–31
Height (cm)	178.2 ± 7.0	161.5–203.0
Body weight (kg)	73.7 ± 7.3	58.3–92.6
Body fat (%)	13.6 ± 2.7	6.7–20.1
Sit‐and‐reach (cm)	48.7 ± 8.6	27.5–79.5
Sit‐and‐reach/height	0.273 ± 0.074	0.163–0.429
Vertical jump (cm)	39.6 ± 9.2	11.7–70.6

*Note*: Data are presented as mean ± SD with ranges. All participants were male professional football players.

Abbreviation: SD, standard deviation.

The study protocol was approved by the Ethics Committee of Juntendo University (approval number 2023‐154), and all participants provided written informed consent before participation.

### Genotyping

Genomic deoxyribonucleic acid (DNA) was obtained from saliva samples using the Oragene® DNA Collection Kit (DNA Genotek). DNA concentration and purity were assessed using a NanoDrop 8000 UV‐Vis Spectrophotometer (Thermo Fisher Scientific) or an Eppendorf BioPhotometer Plus (Eppendorf). Samples were stored at 4°C until analysis.

Genotyping of the COL5A1 rs12722, COL5A1 rs10628678 (insertion/deletion), ACTN3 rs1815739 and ACE rs4341 polymorphisms was performed using TaqMan single‐nucleotide polymorphism (SNP) Genotyping Assays (Applied Biosystems) on a LightCycler® 480 System (Roche Molecular Systems) or QuantStudio® Real‐Time polymerase chain reaction (PCR) System (Thermo Fisher Scientific). The PCR mixture (5 μL total volume) consisted of 2.5 μL of TaqMan® GTXpress™ Master Mix (2×) or TaqMan® Universal Master Mix II (2×), 0.0625 μL of TaqMan® SNP Genotyping Assay mix (40×), 1.4375 μL of sterilized water and 1 μL of genomic DNA (10 ng/μL). Negative controls were included on each plate. Genotypes were determined using LightCycler® 480 (Version 1.5) or QuantStudio® Design and Analysis (Version 1.2) software. From 2023 onwards, genotyping was outsourced to Glissta Co. Ltd. through its healthcare service, IDENSIL, which provides saliva‐based genomic analysis using TaqMan assays. This procedure was applied to 37 soccer players. The outsourced genotyping used the same TaqMan assay design and standardized genotype‐calling procedures. Genotyping procedures followed previously published protocols for COL5A1 polymorphisms using TaqMan® SNP genotyping assays on real‐time PCR platforms (LightCycler 480 or QuantStudio) with duplicate samples and positive and negative controls included in each run [3].

### Statistical analysis

The primary outcome was the occurrence of knee ligament injury (ACL or MCL), and subgroup analysis was performed for ACL injury alone. Genotype distributions were summarized as frequencies and compared between injured and non‐injured players using cross‐tabulation. Global differences across all three genotypes were assessed using Pearson's *χ* ^2^ test; when expected counts were <5, the Fisher–Freeman–Halton exact test was applied. Based on prior biological plausibility, specific genetic contrasts were examined: a recessive model for ACTN3 rs1815739 (XX vs. RR + RX) and a dominant model for COL5A1 rs10628678 (−/−+AGGG/− vs. AGGG/AGGG). Fisher's exact test was used to evaluate categorical differences in these contrasts. Odds ratios (ORs) with 95% confidence intervals (CIs) were calculated for each contrast and for the combined genotype (COL5A1 × ACTN3). When zero cells were present, the Haldane–Anscombe correction (+0.5 to all cells) was applied to stabilize OR and CI estimates. To account for multiple comparisons, *p* values were adjusted using the Benjamini–Hochberg false discovery rate (FDR) approach.

Continuous variables (anthropometric and performance characteristics) were compared between the groups using independent sample *t* tests or one‐way ANOVA, as appropriate. Since this study included all the available players (*n* = 122), an a priori sample size calculation was not performed. A post hoc power analysis, using a two‐proportion framework, indicated that for the overall knee ligament injury outcome (43 cases vs. 79 controls), the study achieved approximately 80% power to detect ORs around 3.0 when the control genotype frequency was approximately 0.30. For ACL‐only analyses (17 cases vs. 105 controls), power was limited, requiring an OR ≥ 4.0 to reach approximately 80% power at similar genotype frequencies.

In addition, a simplified combined risk analysis was performed. Players carrying both the COL5A1 rs10628678 risk allele (AGGG/− or −/−) and ACTN3 rs1815739 XX genotype were classified as the “combined risk” group, whereas all others were considered the reference group. The prevalence of ligament injury between the two groups was compared using chi‐square and Fisher's exact tests, and logistic regression was used to estimate adjusted ORs with 95% CIs.

All analyses were performed using SPSS Statistics, Version 29 (IBM Corp.). Statistical significance was defined as a two‐sided *p* value of <0.05.

## RESULTS

### Participant characteristics

The baseline characteristics of the study participants are presented in Table 1. The study included 122 professional male football players (mean age 22.1 ± 2.2 years), including eight goalkeepers.

### Injury distribution

**Figure 1:**
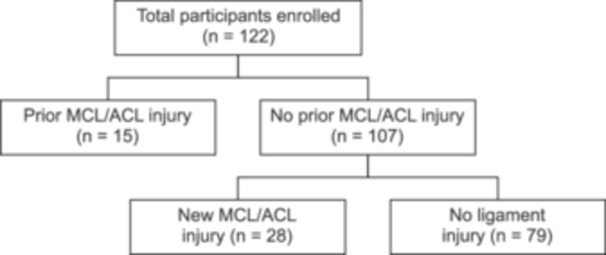
Study flow diagram. In total, 122 professional male football players were enroled in this study. Among them, 15 players had a history of an ACL or MCL injury, whereas 107 had no prior ligament injury. During the observation period, 28 players sustained a new ACL or MCL injury, and 79 players remained free of ligament injuries. ACL, anterior cruciate ligament; MCL, medial collateral ligament.

The study flowchart is illustrated in Figure 1. Of the 122 players, 15 had a history of ACL or MCL injury before joining the team. Among the 107 players without prior ligament injuries, 28 sustained new ACL or MCL injuries during their professional careers, whereas 80 remained free from ligament injuries. Among the players with a history of ligament injury prior to joining the team, only two MCL re‐injuries and no ACL re‐injuries occurred during the observation period. Because of the very small number of recurrent injuries, primary and recurrent injuries were analysed together as overall ligament injuries.

The anthropometric and performance characteristics are summarized in Table S1. No significant differences were observed between the injured (*n* = 43) and non‐injured players (*n* = 79) in terms of height, body composition, flexibility or vertical jump performance.

### Physical characteristics and genotypes

The physical characteristics of the players based on the COL5A1 rs10628678 and ACTN3 rs1815739 genotypes are presented in Table S2. No significant differences were observed among the genotypes in height, body weight, body fat percentage, sit‐and‐reach, sit‐and‐reach/height or vertical jump performance (all *p* > 0.05). However, sit‐and‐reach performance was slightly higher in the COL5A1 rs10628678 −/− group than in other genotypes.

### Genotype distributions and ligament injury

The genotype distributions of COL5A1 rs12722, COL5A1 rs10628678, ACE rs4341, and ACTN3 rs1815739 are summarized in Tables 2 (overall ligament injuries) and 3 (ACL injuries only). All genotype distributions conformed to Hardy–Weinberg equilibrium. For overall ligament injury, COL5A1 rs10628678 showed a significant association in the chi‐square test (*p* < 0.05), with the deletion homozygote (–/–) more frequently observed among injured players compared with carriers of the AGGG allele. Although the difference did not remain significant after FDR adjustment, the distribution suggested a possible trend toward increased susceptibility to ligament injury in this genotype. By contrast, COL5A1 rs12722 and ACE rs4341 showed no significant associations, whereas ACTN3 rs1815739 demonstrated a non‐significant trend in which the XX genotype was more frequent among injured players, consistent with its previously reported relationship with non‐contact injury risk.

**Table 2:** Genotype distributions of COL5A1, ACTN3 and ACE polymorphisms in overall ligament injuries.

Gene	SNP	Genotype	Injured, *n* (%)	Non‐injured, *n* (%)	Total	*p* value (*χ* ^2^)
COL5A1	rs10628678	AGGG/AGGG	4 (13.8)	25 (86.2)	29	**0.016**
		AGGG/−	27 (39.7)	41 (60.3)	68	
		−/−	12 (48.0)	13 (52.0)	25	
COL5A1	rs12722	C/C	30 (38.0)	49 (62.0)	79	0.364
		C/T	13 (32.5)	27 (67.5)	40	
		T/T	0 (0.0)	3 (100.0)	3	
ACE	rs4341	DD	4 (19.0)	17 (81.0)	21	0.164
		ID	24 (42.1)	33 (57.9)	57	
		II	15 (34.1)	29 (65.9)	44	
ACTN3	rs1815739	RR	12 (35.3)	22 (64.7)	34	**0.029**
		RX	16 (26.2)	45 (73.8)	61	
		XX	15 (55.5)	12 (44.4)	27	

*Note*: Data are presented as number of players with percentages in parentheses. *p* values were calculated using the chi‐square (*χ* ^2^) test to compare genotype distributions between injured and non‐injured players. Bold values in the table indicate statistically significant differences (*p* < 0.05).

Abbreviation: SNP, single‐nucleotide polymorphism.

Model‐based contrasts were also tested. For COL5A1 rs10628678, the dominant model (–/–+AGGG/– vs. AGGG/AGGG) indicated a higher risk of overall ligament injury (OR ~ 4.3, 95% CI 1.4–13.4; Fisher *p* = 0.007), although this did not remain significant after FDR correction. For ACTN3 rs1815739, the recessive model (XX vs. RR + RX) showed a significant association with both overall ligament (OR ~ 3.0; 95% CI 1.2–7.2; Fisher *p* = 0.021) and ACL (OR: ~4.0; 95% CI 1.4–11.8; Fisher *p* = 0.013) injuries.

Similar patterns were observed when the analysis was restricted to ACL injuries (Table 3). The COL5A1 rs10628678 deletion homozygote and ACTN3 XX genotype were again more common among ACL‐injured players. However, the limited number of cases reduced statistical power, resulting in wide CIs. Model‐based contrasts reinforced these findings, with ACTN3 XX showing the strongest effect on ACL injury risk under the recessive model.

**Table 3:** Genotype distributions of COL5A1, ACTN3 and ACE polymorphisms in ACL injuries.

Gene	SNP	Genotype	Injured, *n* (%)	Non‐injured, *n* (%)	Total	*p* value (*χ* ^2^)
COL5A1	rs10628678	AGGG/AGGG	1 (3.4)	28 (96.6)	29	0.156
		AGGG/−	11 (16.2)	57 (83.8)	68	
		−/−	5 (20.0)	20 (80.0)	25	
COL5A1	rs12722	C/C	14 (17.7)	65 (82.3)	79	0.245
		C/T	3 (7.5)	37 (92.5)	40	
		T/T	0 (0.0)	3 (100.0)	3	
ACE	rs4341	DD	2 (9.5)	19 (90.5)	21	0.776
		ID	9 (15.8)	48 (84.2)	57	
		II	6 (13.6)	38 (86.4)	44	
ACTN3	rs1815739	RR	4 (11.8)	30 (88.2)	34	**0.025**
		RX	5 (8.2)	56 (91.8)	61	
		XX	8 (29.6)	19 (70.4)	27	

*Note*: Data are presented as number of players with percentages in parentheses. *p* values were calculated using the chi‐square (*χ* ^2^) test to compare genotype distributions between injured and non‐injured players. Bold values in the table indicate statistically significant differences (*p* < 0.05).

Abbreviations: ACL, anterior cruciate ligament; SNP, single‐nucleotide polymorphism.

### Combined genotype analysis

**Figure 2:**
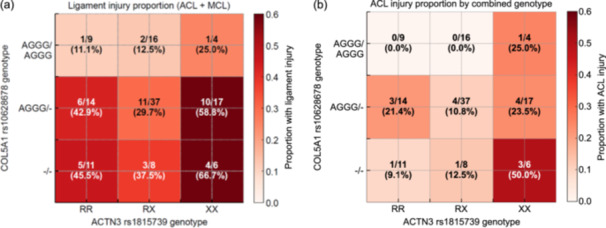
Heatmap of combined genotype associations with ligament injury. (a) Proportion of players with any knee ligament injury (ACL + MCL) based on combined genotypes of COL5A1 rs10628678 and ACTN3 rs1815739. (b) Proportion of players with ACL injury is based only on the same combined genotypes. Each cell shows the number of injured players relative to the total in that genotype category, with percentages in parentheses. A darker red colour indicates a higher proportion of injuries. ACL, anterior cruciate ligament; MCL, medial collateral ligament.

Given these findings, we further examined the interaction between COL5A1 rs10628678 and ACTN3 rs1815739 (Figure 2). Distinct patterns were observed, with the COL5A1 −/− and ACTN3 XX genotypes representing the highest‐risk category. In this subgroup, the proportion of players with ligament injuries exceeded 60% overall and reached 50% among those with ACL injuries.

Forest plot analysis (Figure S1) showed that several combined genotypes exhibited elevated ORs for ligament injury relative to the reference group (AGGG/AGGG–RR), particularly the COL5A1 –/–/ACTN3 XX and AGGG/–/XX combinations, which demonstrated injury rates exceeding 50%.

Although the CIs for these associations crossed unity and none remained significant after FDR correction, the consistent trends across both ACL‐only and ACL + MCL injuries suggest that interactions between ligament‐related (COL5A1) and muscle‐related (ACTN3) genetic factors may meaningfully contribute to susceptibility to severe ligament injury in professional football players. Although some associations were statistically significant in the unadjusted analyses, several did not remain significant after FDR correction and should therefore be interpreted cautiously.

### Combined risk analysis (simplified model)

To aid interpretation, we defined a “combined risk group” as players carrying either the COL5A1 rs10628678 deletion allele (AGGG/– or –/–) or ACTN3 rs1815739 XX genotype. This group consisted of 23 players (18.9% of the cohort). In this subgroup, ligament injury prevalence was 70.7% compared with 29.3% in the remainder (*χ* ^2^ = 8.15, *p* = 0.007). Similarly, ACL injury prevalence was 30.4% versus 10.1%, respectively (*χ* ^2^ = 6.43, *p* = 0.011). Logistic regression using this binary combined‐risk variable confirmed a significant association with ligament injury (OR 3.76, 95% CI 1.46–9.6, *p* = 0.006). These findings indicate that, while single SNP associations were modest, combined genetic profiling captured a subset of players at particularly elevated risk.

## DISCUSSION

This study investigated the association between genetic polymorphisms and knee ligament injuries in professional male football players. The main findings were that the COL5A1 rs10628678 –/– genotype was associated with an increased risk of overall ligament injury (ACL and MCL combined), and that the ACTN3 rs1815739 XX genotype was associated with a higher risk of ACL injury. Notably, when both high‐risk genotypes co‐occurred, the proportion of ACL‐injured players reached 50%, representing the highest‐risk category among the combined genotypes. Although the estimated ORs suggested elevated risks, the 95% CIs crossed unity; thus, statistical significance was not achieved. Nevertheless, the observed patterns were biologically plausible and aligned with previous findings.

Our results are consistent with those of previous studies, highlighting the role of COL5A1 polymorphisms in ligament and tendon injury susceptibility [3, 18, 19, 20]. The COL5A1 gene encodes type V collagen, a regulatory component of type I collagen fibrillogenesis. Altered collagen composition has been implicated in increased tissue fragility and rupture risk. In contrast, ACTN3 and ACE polymorphisms have not been associated with flexibility in Japanese athletes [6]. Kumagai et al. [6], who examined COL5A1 rs12722, reported no association with lower‐limb muscle stiffness or joint range of motion, indicating that this variant may contribute to a risk of injury via mechanisms other than generalized tissue flexibility. Although the COL5A1 rs12722 polymorphism has often been implicated in the stability of mRNA—mediated via 3ʹ‐UTR effects [7], it showed no association in our cohort. In contrast, the rs10628678 variant was associated with a heightened risk of ligament injury. Abrahams et al. have previously reported that the rs10628678 AGGG −/− genotype is associated with an enhancement of sit‐and‐reach performance, indicating greater flexibility [1]. Consistently, our data revealed a trend towards higher flexibility scores in players with this genotype (Online Resource 2), suggesting that an enhanced range of joint motion may represent one mechanism linking rs10628678 with susceptibility to ligament injuries. These findings accordingly support the notion that different COL5A1 polymorphisms may exert their effects via different biological pathways, with rs12722 influencing mRNA stability and rs10628678 potentially modulating connective tissue properties and flexibility.

However, the relationship between joint flexibility and ligament injury risk is complex, as both excessive laxity and excessive stiffness may contribute to injury depending on sport‐specific biomechanical demands. In high‐intensity sports such as football, greater passive joint motion may place increased mechanical demands on dynamic stabilizing structures during cutting, pivoting or landing movements. In this context, the combination of increased connective tissue compliance associated with COL5A1 variants and reduced dynamic muscular stabilization potentially related to the ACTN3 XX genotype may theoretically create a situation in which excessive joint displacement is insufficiently controlled during rapid movements. Although the present study did not directly assess joint biomechanics or neuromuscular control, this interaction between connective tissue properties and muscular stabilization may represent a plausible mechanism linking these genetic variants to ligament injury susceptibility. Further studies integrating genetic, neuromuscular and biomechanical assessments are needed to clarify these potential mechanisms.

The current findings are also consistent with reports that the ACTN3 rs1815739 XX genotype is associated with higher rates of non‐contact injuries in athletes [23]. The ACTN3 gene encodes α‐actinin‐3, a structural protein expressed in fast‐twitch muscle fibres [22]. Loss of α‐actinin‐3 expression in XX individuals compromises explosive muscle force and may reduce dynamic joint stabilization, thereby increasing ACL injury risk [13]. Our data extend these observations by showing that ACTN3 XX status may synergize with COL5A1 variants to further elevate the risk. Notably, when both risk alleles were considered simultaneously as a combined risk variable, players carrying the COL5A1 deletion allele together with the ACTN3 XX genotype exhibited a three‐fold increase in ligament injury risk, as confirmed by cross‐tabulation (60.9% vs. 29.3% for overall injury; 30.4% vs. 10.1% for ACL) and logistic regression OR 3.76, 95% CI 1.46–9.6). Approximately 19% of the cohort fell into this high‐risk category, underscoring the potential clinical utility of combined genetic profiling for identifying a substantial subgroup of players at elevated risk. To our knowledge, this is the first study to demonstrate that both ligament‐related genetic characteristics (COL5A1) and muscle function‐related genetic characteristics (ACTN3) contribute to injury risk in a complementary manner.

The null finding for ACE is informative. Triangulating structural risk (COL5A1) and neuromuscular (ACTN3) signals against a vascular re‐modelling candidate (ACE), which showed no association, enabled us to delineate the potential range of effect sizes along the functional axis. This observation reinforces that the observed combined‐risk pattern identified in this study is not merely an artefact of including any polymorphism.

From a clinical perspective, although MCL injuries are more common in football players, ACL injuries are less frequent, more severe and result in prolonged absence from play [5, 8]. Therefore, identifying athletes with a genetically increased risk of ACL injury holds considerable relevance. The novelty of our study lies in its exclusive focus on professional football players and the evaluation of combined genotypes, which have not been previously reported. Our additional findings suggest that simplified combined risk models may pragmatically stratify players for targeted preventive interventions. A once‐in‐a‐lifetime genetic test provides unmodifiable information [2] that can guide targeted interventions. Athletes with high‐risk genotypes may benefit from prioritization for preventive programs such as neuromuscular training, proprioception exercises or individualized load management, potentially reducing their elevated risk.

The broader genetic literature emphasizes that injury susceptibility is polygenic and influenced by multiple pathways, including extracellular matrix metabolism and inflammatory responses [19, 20]. Although our analysis was limited to COL5A1, ACTN3 and ACE, future studies should consider polygenic risk scores that integrate multiple loci. International multicentre collaborations will also be essential to increase statistical power and evaluate gene–environment interactions. Moreover, prospective designs that combine genetic data with biomechanical and imaging assessments could clarify the mechanisms linking genotypes to tissue failure.

This study had some limitations. First, although the sample size was modest, it represents one of the largest genetic cohorts that focuses exclusively on professional football players, enhancing its relevance within this population. Second, the study was limited to male Japanese athletes from a single team; thus, the generalizability of the findings to other populations or female players remains uncertain. Notably, in female football players, COL5A1 variants are associated with a risk of ACL injury [16], indicating that sex‐specific genetic effects may warrant further investigation. Third, only a limited set of candidate polymorphisms was analysed, and non‐genetic risk factors, such as neuromuscular control, joint biomechanics and extrinsic load, were not assessed. In addition, although some associations were statistically significant in the unadjusted analyses, several did not remain significant after FDR correction and should therefore be interpreted cautiously. Accordingly, the simplified combined genotype model should be considered exploratory and hypothesis‐generating rather than a definitive predictive model. Further validation in larger independent cohorts and in diverse athletic populations will be necessary to confirm these findings.

In summary, both global distribution analyses and model‐based contrasts suggested that COL5A1 rs10628678 and ACTN3 rs1815739 may be associated with knee ligament injury risk in professional football players. Genetic information should not be interpreted deterministically, as ligament injury risk is influenced by multiple intrinsic and extrinsic factors. Even athletes without the examined variants may sustain injuries due to biomechanical or training‐related factors, whereas the presence of risk‐associated variants does not necessarily lead to injury. Therefore, genetic testing, if applied in the future, should be interpreted cautiously and integrated with established injury prevention strategies. Larger multicentre studies are needed to validate these findings and to determine whether integrating ligament‐related and muscle‐related genetic factors can contribute to individualized injury prevention strategies.

## CONCLUSION

COL5A1 and ACTN3 polymorphisms may be associated with knee ligament injury susceptibility in professional football players. In particular, the combined COL5A1 rs10628678 –/– and ACTN3 rs1815739 XX genotypes may represent a higher‐risk genetic profile for ACL injury. Genetic testing, performed once in a lifetime, may provide valuable information to support the implementation of individualized preventive strategies for high‐risk athletes when interpreted alongside established biomechanical and training‐related risk factors. Larger multicentre studies are required to validate these findings.

## AUTHOR CONTRIBUTIONS

All authors contributed to the study design, data collection, analysis and manuscript preparation. All the authors have read and approved the final version of the manuscript.

## CONFLICT OF INTEREST STATEMENT

The authors declare no conflicts of interest.

## ETHICS STATEMENT

This study was approved by the Ethics Committee of Juntendo University (approval number 2023‐154). All the participants provided written informed consent. Informed consent was obtained from all participants. The participant consented to the submission of the case report to the journal.

## Supporting information

ESM_1.

ESM_2.

**Online resource 3: Forest plots of combined genotype associations with ligament injury.** (A) Odds ratios (ORs) for overall knee ligament injury (ACL + MCL) based on combined genotypes of COL5A1 rs10628678 and ACTN3 rs1815739. (B) Odds ratios for ACL injuries only. The reference category for both plots is AGGG/AGGG – RR. Dots represent ORs, and horizontal lines indicate 95% confidence intervals. The dashed vertical line indicates an OR of 1.

## Data Availability

The datasets generated and/or analysed during the current study are available from the corresponding author upon reasonable request.
