# Supplementary material for: Collagen Type V alpha 1 chain and alpha‐actinin‐3 variants predict knee ligament injury risk in professional football players
Source: J Exp Orthop. 2026 Apr 20;13(2):e70724. doi: 10.1002/jeo2.70724 (PMC13093292; doi:10.1002/jeo2.70724)
Supplement: Supplementary file 1 — ESM_1. [file JEO2-13-e70724-s002.docx]

**Online Resource 1. Anthropometric and physical performance characteristics in players with and without knee ligament injury**

| **Variable** | **Non-injured (n=79)** | **Injured (n=43)** | **p-value** |
| --- | --- | --- | --- |
| Height (cm) | 178.9 ± 7.1 | 177.0 ± 6.8 | n.s. |
| Body weight (kg) | 74.2 ± 7.3 | 72.8 ± 7.3 | n.s. |
| Body fat (%) | 13.5 ± 2.8 | 13.9 ± 2.5 | n.s. |
| Sit-and-reach (cm) | 48.1 ± 8.5 | 49.8 ± 8.7 | n.s. |
| Sit-and-reach / Height | 0.269 ± 0.045 | 0.282 ± 0.050 | n.s. |
| Vertical jump (cm) | 40.3 ± 9.7 | 38.4 ± 8.2 | n.s. |

Values are presented as mean ± standard deviation. An independent samples t-test was used to compare groups. No significant differences were observed between variables.
