# Supplementary material for: Collagen Type V alpha 1 chain and alpha‐actinin‐3 variants predict knee ligament injury risk in professional football players
Source: J Exp Orthop. 2026 Apr 20;13(2):e70724. doi: 10.1002/jeo2.70724 (PMC13093292; doi:10.1002/jeo2.70724)
Supplement: Supplementary file 2 — ESM_2. [file JEO2-13-e70724-s003.docx]

**Online Resource 2. Physical characteristics of the players based on the COL5A1 rs10628678 and ACTN3 rs1815739 genotypes**

|  | **COL5A1 rs10628678** | | | **ACTN3 rs1815739** | | |
| --- | --- | --- | --- | --- | --- | --- |
| **Variable** | **AGGG/AGGG (n=29)** | **AGGG/- (n=68)** | **-/- (n=25)** | **RR (n=34)** | **RX (n=61)** | **XX (n=27)** |
| Height (cm) | 179.0 ± 8.6 | 177.9 ± 6.4 | 178.1 ± 6.8 | 179.0 ± 7.8 | 178.2 ± 6.7 | 177.2 ± 6.7 |
| Body weight (kg) | 73.8 ± 6.9 | 73.5 ± 7.3 | 74.2 ± 8.1 | 74.2 ± 8.4 | 73.5 ± 6.6 | 73.5 ± 7.5 |
| Body fat (%) | 12.9 ± 3.0 | 13.8 ± 2.5 | 12.7 ± 2.9 | 13.1 ± 2.5 | 13.6 ± 3.0 | 12.7 ± 3.2 |
| Sit-and-reach (cm) | 48.7 ± 7.2 | 48.4 ± 8.5 | 49.5 ± 10.6 | 47.5 ± 9.9 | 50.1 ± 8.1 | 46.9 ± 7.7 |
| Sit-and-reach / Height | 0.272 ± 0.06 | 0.272 ± 0.05 | 0.278 ± 0.05 | 0.265 ± 0.05 | 0.281 ± 0.05 | 0.266 ± 0.04 |
| Vertical jump (cm) | 40.9 ± 10.0 | 38.5 ± 8.2 | 39.5 ± 9.6 | 40.7 ± 8.3 | 39.3 ± 8.9 | 39.6 ± 9.1 |

Values are presented as mean ± standard deviation. No significant differences were detected between the genotypes (ANOVA, all *p* > 0.05). The Sit-and-reach test scores tended to be higher in the rs10628678 –/– group.
