# Supplementary material for: Collagen Type V alpha 1 chain and alpha‐actinin‐3 variants predict knee ligament injury risk in professional football players
Source: J Exp Orthop. 2026 Apr 20;13(2):e70724. doi: 10.1002/jeo2.70724 (PMC13093292; doi:10.1002/jeo2.70724)

Online resource 3: Forest plots of combined genotype associations with ligament injury.

(A)

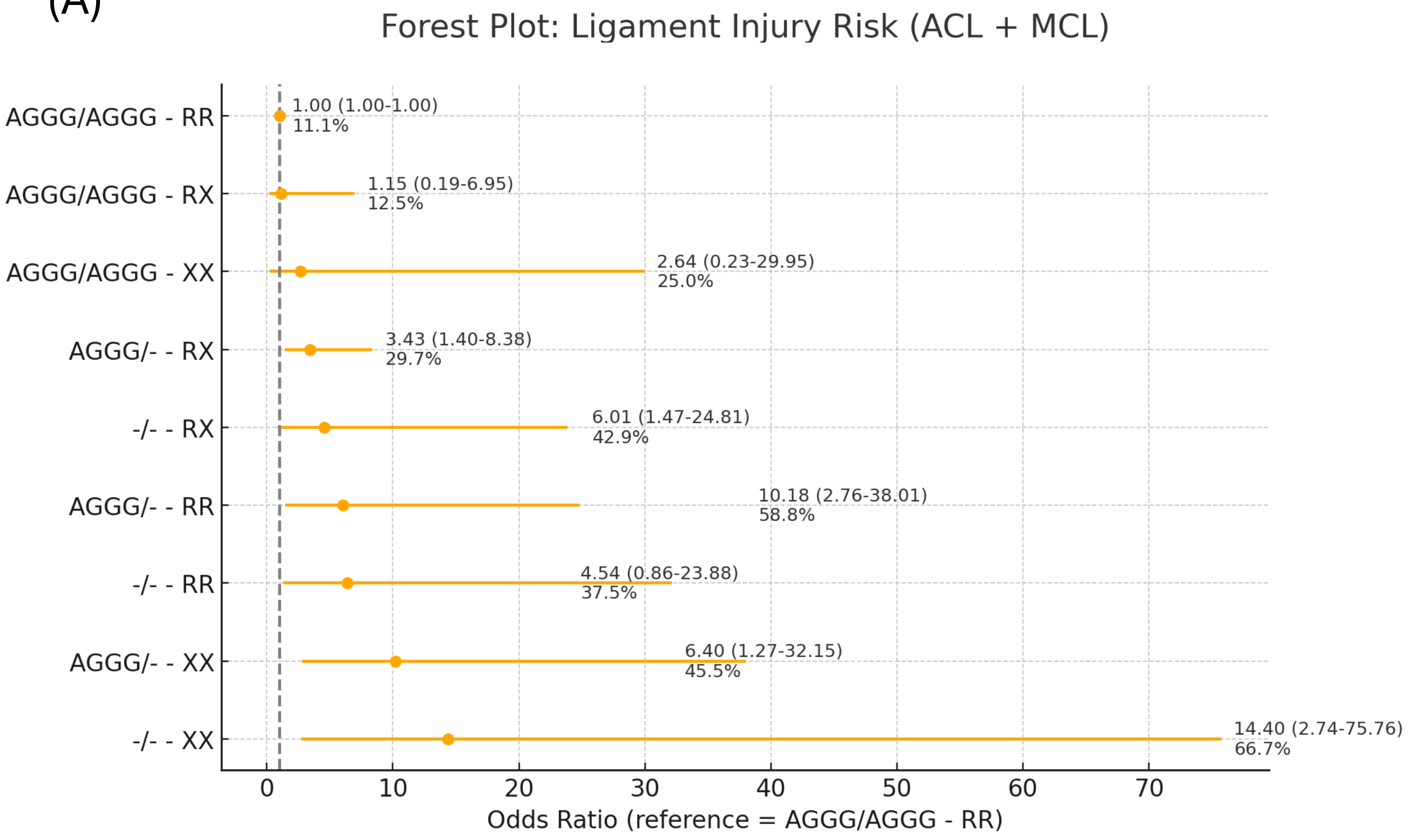

(B)

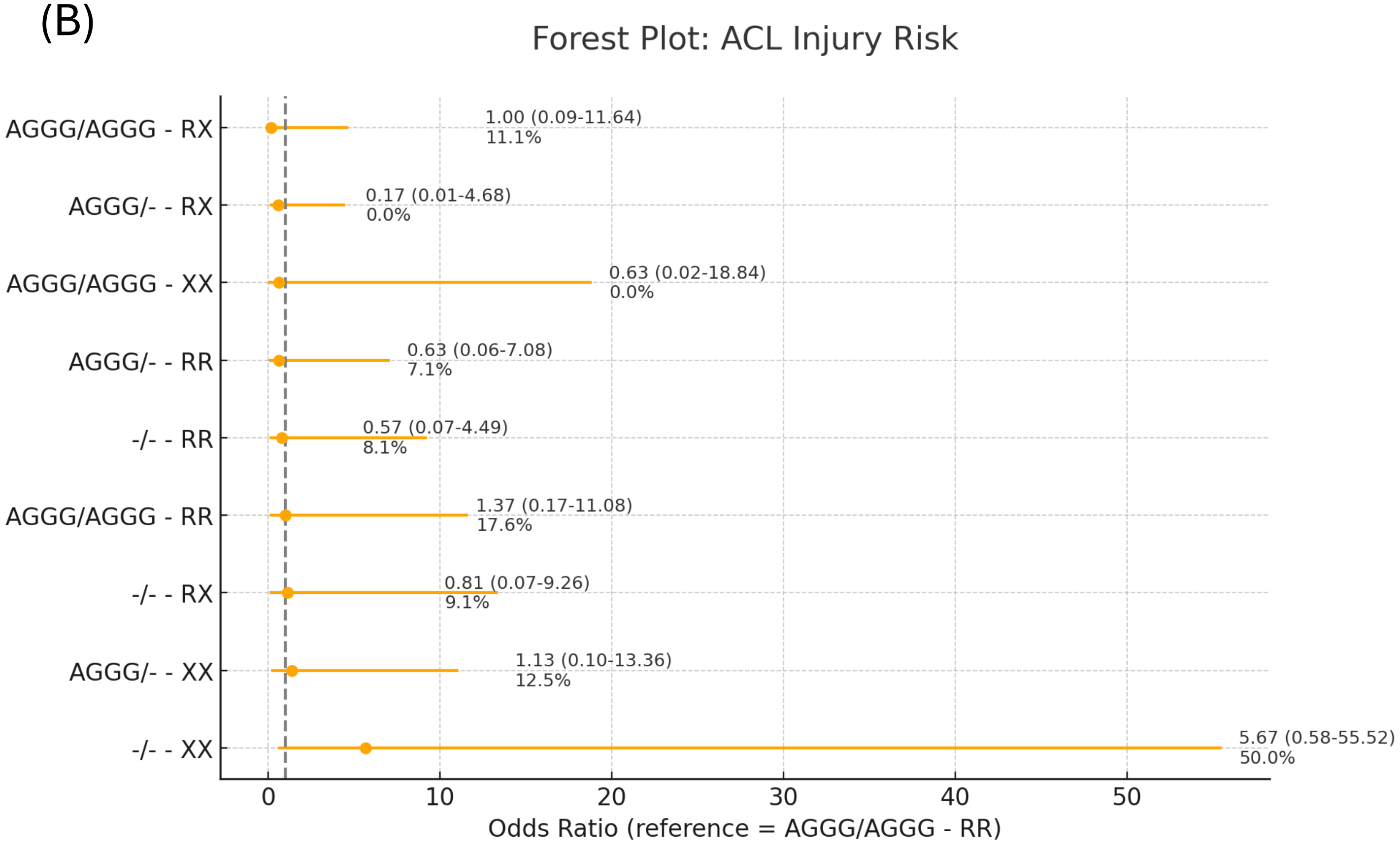

Supplement: Supplementary file 3 — Online resource 3: Forest plots of combined genotype associations with ligament injury. (A) Odds ratios (ORs) for overall knee ligament injury (ACL + MCL) based on combined genotypes of COL5A1 rs10628678 and ACTN3 rs1815739. (B) Odds ratios for ACL injuries only. The reference category for both plots is AGGG/AGGG – RR. Dots represent ORs, and horizontal lines indicate 95% confidence intervals. The dashed vertical line indicates an OR of 1. [file JEO2-13-e70724-s001.pdf]
